# Diagnostic yield, indications, and outcomes of cranial imaging in AML patients admitted for intensive induction or consolidation chemotherapy: a single-center experience

**DOI:** 10.1007/s00277-023-05178-6

**Published:** 2023-03-22

**Authors:** Sebastian E. Koschade, Jan A. Stratmann, Pia S. Zeiner, Fabian Finkelmeier, Jörg Chromik, Björn Steffen, Hubert Serve, Christian H. Brandts, Olivier Ballo

**Affiliations:** 1 Department of Medicine, Hematology/Oncology, University Hospital Frankfurt, Goethe University, Frankfurt Am Main, Germany; 2 Dr. Senckenberg Institute of Neurooncology, University Hospital Frankfurt, Goethe University, Frankfurt Am Main, Germany; 3 University Cancer Center Frankfurt (UCT), University Hospital Franfurt, Goethe University, Frankfurt Am Main, Germany; 4 Department of Medicine, Gastroenterology, Hepatology and Endocrinology, University Hospital Frankfurt, Goethe University, Frankfurt Am Main, Germany

**Keywords:** AML, Cranial imaging, Bleeding, Infection, Intensive chemotherapy

## Abstract

Cranial imaging (CI) is a widely used diagnostic procedure, especially in acute myeloid leukemia (AML) patients with suspected bleeding or infection. However, common clinical decision rules to guide CI do not apply to AML patients and the diagnostic yield and outcomes of CI for AML patients are largely unknown. We retrospectively evaluated all CI from newly diagnosed non-promyelocytic AML patients receiving intensive induction or consolidation chemotherapy between 2007 and 2019 for imaging indications, diagnostic yield, and consequences. A total of 110 of 462 patients (24%) received CI for 152 imagings in distinct clinical situations. Forty-four patients (40%) had at least one new and acute pathological finding. Main indication was focal neurologic deficit, craniocerebral trauma, and suspected cerebral hypertension. The most common new finding was intracranial hemorrhage (13% of all imagings), followed by sinusitis (9%). CI led to therapy change in 21 patients. There were no clear associations between indications, laboratory values, and a positive imaging. Positive imaging was associated with adverse overall survival. Our study suggests that the overall rate of ordered CI was appropriate and that CI should generally be performed at a low threshold. A systematized approach to CI may further increase diagnostic yield but is complicated by variable clinical presentation.

## Introduction

Acute myeloid leukemia (AML) is an aggressive malignant hematopoietic disease and the most frequent acute leukemia in adults [1]. Curative treatment mainly consists of high-intensity induction chemotherapy followed by either consolidation chemotherapy or primary allogeneic stem cell transplantation [1]. Patients regularly present with or develop severe cytopenia both due to the underlying disease itself with variable degree of bone marrow failure and due to the administered cytotoxic chemotherapy. This predisposes patients to infections and bleeding complications and often requires lengthy hospital stays for curative treatment administration, close surveillance, and careful management of side effects and complications.

Cranial computed tomography (CCT) and other cranial imaging (CI) modalities have become widely available and the number of CI scans has steadily increased [2]. Radiation dose reductions, the perceived low risk of radiation exposure in the context of a highly aggressive malignant disease such as AML, and the risk of bleeding and infection contribute to a low threshold for ordering CCTs. However, commonly employed clinical decision rules to guide CI do not apply to AML patients undergoing therapy [3, 4] and the diagnostic yield of CI, primary indications for ordering CI, and radiologic findings and therapeutic consequences have not yet been described systematically for newly diagnosed AML patients undergoing intensive induction or consolidation chemotherapy. There is little evidence for any specific guideline for ordering CI in AML patients [5–7].

Given the lack of available data, we conducted a single-center retrospective study and analyzed indications, associated clinical signs and symptoms, radiologic results, and therapeutic consequences of CI in AML patients that were treated at our clinic from 2007 to 2019. Secondary objective was the determination of impact of positive imaging upon overall survival of AML patients.

## Methods

### Patients

All patients newly diagnosed with AML (excluding APL) according to WHO criteria [8] who underwent intensive induction chemotherapy or consolidation chemotherapy between 2007 and 2019 at our institution were retrospectively included. Patients were identified as detailed below. The screening period consisted of the duration of the hospital stays. Study group and screening period were predefined. Standard induction chemotherapy was cytarabine 100 mg/m^2^ administered continuously for 7 days together with daunorubicin 60 mg/m^2^ for 3 days (7 + 3). Patients younger than 60 years received either a second induction therapy cycle of 7 + 3 if they had achieved bone marrow blast clearance on day 15 after start of induction therapy, or they received a salvage induction therapy cycle consisting of cytarabine 3000 mg/m^2^ every 12 h for 3 days and mitoxantrone 10 mg/m^2^ for 3 days (HAM) if blast clearance was not achieved on day 15 [9]. Patients 60 years or older received a second induction therapy cycle with HAM (with reduced cytarabine dose of 1000 mg/m^2^) only if they had not achieved bone marrow blast clearance on day 15. Consolidation chemotherapy was generally administered to patients younger than 60 years who achieved complete remission as three courses of high-dose cytarabine (3 g/m^2^ intravenously over 3 h per q12 hours on days 1–3) not earlier than 1 week after attaining CR (HDAC) [10]. Patients older than 60 years generally received two courses of intermediate-dose cytarabine (1 g/m^2^ intravenously over 3 h per q12 hours on 3 days (IDAC) [11]. Allogeneic hematopoietic stem cell transplantation instead of consolidation chemotherapy was recommended for patients with intermediate or adverse risk according to ELN risk group stratification [10]. Standard antimicrobial prophylaxis consisted of posaconazole and levofloxacin [12, 13]. Laboratory values, including hemoglobin (Hb), platelet count, activated partial thromboplastin time, international normalized ratio, and C-reactive protein, were monitored routinely. Thresholds used for transfusion were Hb < 8.0 g/dl (2007–08/2014)/Hb ≤ 7.0 g/dl (after 08/2014) and/or platelet count < 10/nl, except for febrile patients (Hb ≤ 8.0 g/dl and/or platelet count < 20/nl). The platelet transfusion threshold remained consistent throughout the entire time period. Pooled random donor platelet concentrates were routinely administered.

Consent to anonymized publication and patient data were provided after approval by the local ethics committee (ref. nr. UCT-71–2020). All procedures followed were in accordance with the ethical standards of the responsible committee on human experimentation (institutional and national) and with the 2013 Declaration of Helsinki. Consecutively admitted non-APL AML patients were identified from the digital cancer registry of the University Cancer Center Frankfurt by database search on ICD-10 code and date of diagnosis and annotated based on archived medical records and manual chart review as described before [14]. Results from all inhouse CI studies for this patient cohort were retrieved from the medical records and manually annotated. Reported imaging results had been validated by a senior attending radiologist. The clinical trigger for CI was not pre-specified; reasons for performing CI were extracted from the patients’ records. Neurologic symptoms and indications for imaging were grouped based on all available information in the patients’ records into (1) acute focal neurologic deficits, (2) cerebral hypertension (headache, nausea/emesis, and altered mental state), (3) meningitic/encephalitic syndrome (fever, headache, and nuchal rigidity), (4) craniocerebral trauma, (5) encephalopathic syndrome (psychomotor retardation and altered mental state), (6) isolated headache (severe, prolonged, or of new characteristics to the patient), and (7) suspected extracerebral infection. Among results, only uncomplicated sinusitis and thrombosis of the internal jugular vein were regarded as minor pathologies. A high degree of suspicion for intracranial hemorrhage and infection was maintained throughout the study period and patients with accidental falls, headaches, reduced vigilance, altered mental state, focal neurological findings, or suspected infection received prompt CI. Inhouse CT and magnetic resonance imaging (MRI) modalities were available 24/7 during the entire study period.

### Statistical analysis

R 4.0.3 [15] and ggplot2 3.3.2 [16] were used for statistical analyses, data reporting, and plotting. Differences in proportion and other numerical variables between groups were tested using Chi^2^ test and Mann–Whitney *U* test. Overall survival was estimated using the Kaplan–Meier method and survival between patient groups was compared with the log-rank test. The reverse-KM method was used to estimate median follow-up time. Cox proportional hazard regression analysis was used for multivariate survival analysis. A *P* value < 0.05 was considered as statistically significant.

## Results

### Baseline characteristics of AML patients

**Fig. 1:**
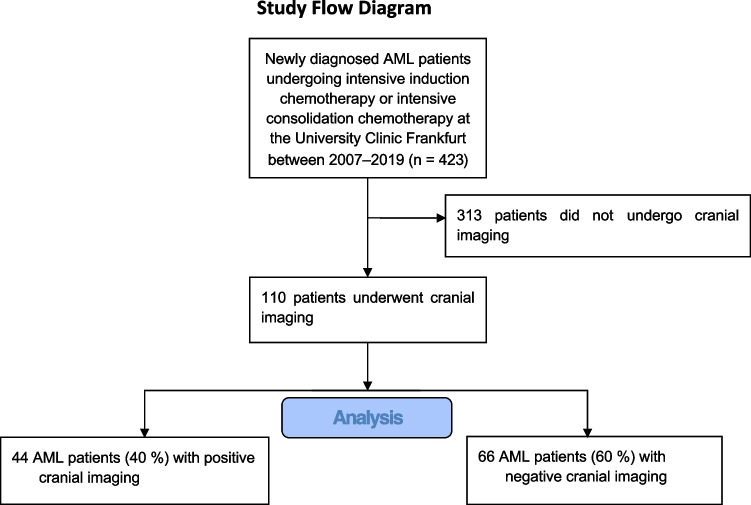
Study flow diagram

We identified 462 newly diagnosed AML patients who underwent intensive induction or consolidation chemotherapy between 2007 and 2019 and 110 patients who received CI during their hospital stay for induction or consolidation chemotherapy (Table 1 and Fig. 1). In total, 152 CIs were performed in these 110 patients (follow-up imaging excluded), because some patients received multiple non-follow-up imaging for separate indications. Forty-eight of 152 CIs (32%) revealed an acute new pathological finding (“positive imaging”), and CI was positive for an acute new pathological finding in 44 AML patients (40%). CI revealed a major pathology (e.g., bleeding, infarction cerebral hypertension, and fracture) in 33 AML patients and a minor acute new pathology (such as uncomplicated sinusitis) in 11 AML patients. The study group is described in Table 1. AML patients with positive imaging were more frequently female (*P* = 0.006). Median age was 62 years (range, 22–78 years) in AML patients with positive imaging and 55 years (range, 20–85 years) in AML patients with negative imaging (*P* = 0.03). Patients with positive and negative imaging did not differ between AML risk groups according to the European Leukemia Net (ELN) recommendations from 2010 [10]. Median follow-up time were 64 months (95% confidence interval, 53–75 months).

**Table 1:** Baseline characteristics

		All patients (*n* = 110)	Positive imaging (*n* = 44)			Negative imaging (*n* = 66)	*P* value*
				Major pathology (*n* = 33)	Minor pathology (*n* = 11)		
Gender	Female	51	28 (64%)	22 (67%)	6 (55%)	23 (35%)	0.006
	Male	59	16 (36%)	11 (33%)	5 (45%)	43 (65%)	
Age at diagnosis, median (range), years		59 (20–85)	62 (22–78)	62 (22–78)	59 (38–73)	55 (20–85)	0.03
WHO classification	AML with recurrent genetic abnormalities	53	24 (55%)	17 (52%)	7 (64%)	29 (44%)	0.37
	AML with dysplasia-related changes	12	3 (7%)	3 (9%)	0 (0%)	9 (14%)	0.42
	Therapy-related AML	1	1 (2%)	1 (3%)	0 (0%)	0 (0%)	0.84
	AML, not otherwise specified	44	16 (36%)	12 (36%)	4 (36%)	28 (42%)	0.66
ELN 2010	Favorable	27	10 (23%)	6 (18%)	4 (36%)	17 (26%)	0.89
	Intermediate-I	40	15 (34%)	11 (33%)	4 (36%)	25 (38%)	0.84
	Intermediate-II	23	12 (27%)	9 (27%)	3 (27%)	11 (17%)	0.27
	Adverse	18	6 (14%)	6 (18%)	0 (0%)	12 (18%)	0.71
	Not classified	2	1 (2%)	1 (3%)	0 (0 %)	1 (2%)	1

Count data is shown unless indicated otherwise

^*^Differences between patients with positive imaging and with negative imaging were tested

### Main indications for cranial imaging and associated findings

The main indications for obtaining imaging were acute focal neurologic deficits and craniocerebral trauma in 31 (20%) scans each, followed by suspected cerebral hypertension in 24 (16%) scans, isolated headache in 23 (15%) scans, encephalopathic syndrome in 14 (9%) scans, and suspected extracerebral infection in 10 (7%) scans (Table 2). There was no specific indication or type of neurologic syndrome that showed a statistically significant association with a positive finding of acute new pathology. Likewise, craniocerebral trauma due to an inhouse hospital fall was not associated with a positive finding on CI (Table 2). Laboratory values at the time of imaging also showed no significant association with a positive finding on CI (Table 2).

**Table 2:** Indications for cranial imaging, preceding events/neurological deficits, pathological findings, and consequences

		All	Positive imaging			Negative imaging	*P* value*
				Major pathology	Minor pathology		
Number of cranial imaging		**152**	**48 (32%)**	**37 (24%)**	**11 (7%)**	**104 (68%)**	
Patients		110	44 (40%)	33 (30%)	11 (10%)	66 (60%)	
Treatment phase	Prior to induction therapy	24 (16%)	10 (21%)	6 (16%)	4 (36%)	14 (13%)	0.36
	Induction therapy	113 (74%)	34 (71%)	27 (73%)	7 (64%)	79 (76%)	0.64
	Consolidation therapy	15 (10%)	4 (8%)	4 (11%)	0 ( 0%)	11 (11%)	0.89
Laboratory values	CRP (mg/dl)	6.4 (2.7–12.6)	8.3 (3.0–15.2)	7.5 (2.8–14.9)	10.4 (7.8–20.7)	5.7 (2.3–12.0)	0.16
	Leukocytes (/nl)	0.8 (0.3–6.2)	0.9 (0.5–16)	0.7 (0.5–3.8)	2.3 (0.6–39.9)	0.7 (0.2–5.0)	0.17
	Hemoglobin (g/dl)	8.5 (7.7–9.6)	8.5 (8.0–9.4)	8.5 (8.0–9.4)	8.4 (8.3–9.6)	8.5 (7.5–9.7)	0.56
	Thrombocytes (/nl)	23 (12–42)	33 (14–70)	25 (13–62)	42 (24–102)	21 (12–35)	0.054
	TPZ (quick) (%)	77 (67–89)	76 (64–89)	77 (67–90)	67 (58–85)	78 (70–89)	0.40
	INR	1.2 (1.1–1.3)	1.2 (1.1–1.4)	1.2 (1.1–1.3)	1.3 (1.1–1.5)	1.2 (1.1–1.3)	0.38
	aPTT (sec)	31 (28–38)	34 (28–41)	31 (28–39)	42 (36–45)	30 (28–37)	0.07
Primary indication for cranial imaging	Acute focal neurologic deficits	31 (20%)	9 (19%)	7 (19%)	2 (18%)	22 (21%)	0.90
	Cerebral hypertension	24 (16%)	10 (21%)	10 (27%)	0 (0%)	14 (13%)	0.36
	Meningitic/encephalitic syndrome	6 (4%)	1 (2%)	1 (3%)	0 (0%)	5 (5%)	0.72
	Craniocerebral trauma	31 (20%)	10 (21%)	10 (27%)	0 (0%)	21 (20%)	1
	Encephalopathic syndrome	14 (9%)	5 (10%)	2 (5%)	3 (27%)	9 (9%)	0.96
	Isolated headache	23 (15%)	5 (10%)	3 (8%)	2 (18%)	18 (17%)	0.39
	Suspected extracerebral infection	10 (7%)	3 (6%)	2 (5%)	1 (9%)	7 (7%)	1
	Other	10 (7%)	3 (6%)	2 (5%)	1 (9%)	7 (7%)	1
	Not specified	3 (2%)	2 (4%)	0 (0%)	2 (18%)	1 (1%)	0.49
Modality	CCT	140 (92%)	45 (94%)	34 (92%)	11 (100%)	95 (91%)	0.85
	cMRT	12 (8%)	3 (6%)	3 (8%)	0 (0%)	9 (9%)	0.85
Imaging with contrast agent		46 (30%)	19 (40%)	12 (32%)	7 (64%)	27 (26%)	0.13
Consequences	None	111 (73%)	15 (31%)	6 (16%)	9 (82%)	96 (92%)	< 0.0001
	Control imaging	19 (13%)	11 (23%)	10 (27%)	1 (9%)	8 (8%)	0.01
	Therapy change	15 (10%)	15 (31%)	14 (38%)	1 (9%)	0 (0%)	< 0.0001
	Surgery	6 (10%)	6 (13%)	6 (16%)	0 (0%)	0 (0%)	0.001
	Lumbar puncture	1 (1%)	1 (2%)	1 (3%)	0 (0%)	0 (0%)	0.69

Count data is shown unless indicated otherwise. The number of cranial imagings (bold) indicates the total. For laboratory values, median and inter-quartile range (IQR) are shown

^*^Differences between patients with positive imaging and with negative imaging were tested

### Pathologies in cranial imaging and clinical consequences

Out of 48 positive imagings, the most frequently diagnosed new and acute entity was intracranial hemorrhage in 20 scans (42% of all positive imagings) consisting of intracerebral bleeding in 12 scans (25%), subdural hematoma in 7 scans (15%), subarachnoid bleeding in 4 scans (8%), and epidural bleeding in 1 scan (2%), followed by sinusitis in 15 scans (31%) and galea hematoma in 6 scans (13%) (Table 3). Infarction was found in 3 scans (6%), cerebral edema and fracture in 2 scans each (4%). Tumor, thrombosis, phlegmon, and posterior reversible encephalopathy syndrome were isolated findings in 1 scan (2%) each. In 5 cases, primary CI highlighted unclear pathologies with indication for follow-up diagnostics.

**Table 3:** New pathological findings of cranial imagings

	*N*	% of positive imaging	% of all imaging
ICH (any)	20	42%	13%
–ICB	12	25%	8%
–Subdural hematoma	7	15%	5%
–SAB	4	8%	3%
–Epidural bleeding	1	2%	1%
Sinusitis	15	31%	10%
Galea hematoma	6	13%	4%
Cerebral edema	2	4%	1%
Ischemic stroke	3	6%	2%
Fracture	2	4%	1%
Tumor	1	2%	1%
Thrombosis IJV	1	2%	1%
Phlegmon	1	2%	1%
PRES	1	2%	1%
Other	3	6%	2%
Unclear pathology, follow-up diagnostic req.	5	10%	3%

*ICH*, intracranial hemorrhage; *ICB*, intracerebral bleeding; *SAB*, subarachnoid bleeding; *IJV*, internal jugular vein; *PRES*, posterior reversible encephalopathy syndrome; *req.*, required

Some patients had multiple findings, categories are not exclusive

Of the 48 positive imagings, 15 scans (31%) directly led to a change in therapy, 6 scans (13%) led to surgical intervention, 11 scans (23%) resulted in repeat/follow-up imaging, and 15 scans (31%) did not result in any follow-up procedure or change of therapy despite positive finding. Out of the 104 negative imagings, 8 scans (8%) led to a follow-up imaging (described below) and 96 scans (92%) resulted in no further follow-up.

Among the chronic or old findings on imaging, chronic sinusitis was the most frequent in 21 scans (14% of all imaging), residual cerebral defects were noted in 20 scans (13%), followed by calcifications (13 scans, 9%), arteriosclerosis or microvasculatory changes (9 scans, 6%), leukoencephalopathy (2 scans, 2%), and others (Table 4). Pre-existing cranial abnormalities were not associated with new pathological findings upon imaging (Table 4). No direct changes in therapy were made due to these chronic or old findings.

**Table 4:** Chronic/old pathological findings of cranial imaging

	All	Positive imaging	Negative imaging	*P* value*
Chronic sinusitis	21 (14%)	5 (3%)	16 (10%)	0.57
Residual cerebral defects	20 (13%)	5 (3%)	15 (10%)	0.67
Calcifications	13 (9%)	5 (3%)	8 (5%)	0.81
Arteriosclerosis/microvasculatory changes	9 (6%)	2 (1%)	7 (5%)	0.80
Leukoencephalopathy	2 (1%)	1 (1%)	1 (1%)	1
Other	13 (9%)	3 (2%)	10 (7%)	0.71

Some patients had multiple findings, categories are not exclusive. Positive/negative imaging refers to acute new pathologies as described in Tables 2 and 3. Percentages relate to the total number of imaging

### Diagnostic yield of repeated cranial imaging after negative first imaging

We identified 8 AML patients with initial negative imaging who received follow-up CI despite a negative first CI. In all cases, the reason to order repeat imaging was persisting clinical suspicion of an acute intracranial pathology despite negative first (non-contrast enhanced) CCT imaging. In two patients, acute new pathological findings were identified with cMRT (subdural hygroma, ischemic stroke); in both cases, this led to a change in management (Table 5).

**Table 5:** Indications, findings, and consequences of second cranial imaging after negative first imaging

		All	Positive imaging	Negative imaging	*P* value*
Patients		8	2	6	
Number of cranial imagings		**8**	**2**	**6**	
Modality	CCT	3 (38%)	0	3 (50%)	0.67
	cMRT	5 (63%)	2 (100%)	3 (50%)	0.67
Imaging with contrast agent		7 (88%)	2 (100%)	5 (83%)	1
Primary indication for cranial imaging	Neurologic deficit	3 (38%)	1 (50%)	2 (33%)	1
	Headache	1 (13%)	1 (50%)	0	0.54
	Fall	1 (13%)	0	1 (17%)	1
	Seizure	1 (13%)	0	1 (17%)	1
	Other	1 (13%)	0	1 (17%)	1
	Not specified	1 (13%)	0	1 (17%)	1
Neurologic deficits	Altered mental state	2 (25%)	0	2 (33%)	1
	Visual deficit	1 (13%)	0	1 (17%)	1
	Paresis	2 (25%)	1 (17%)	1 (17%)	1
Results	Ischemic stroke	1 (13%)	1 (50%)	0	0.54
	Subdural hygroma	1 (13%)	1 (50%)	0	0.54
Consequences	None	6	0	6 (100%)	0.06
	Therapy/management change	2	2 (100%)	0	0.06

Count data is shown unless indicated otherwise. The number of cranial imagings (bold) indicates the total

^*^Differences between patients with positive imaging and with negative imaging were tested

### Impact of positive findings on overall survival

**Fig. 2:**
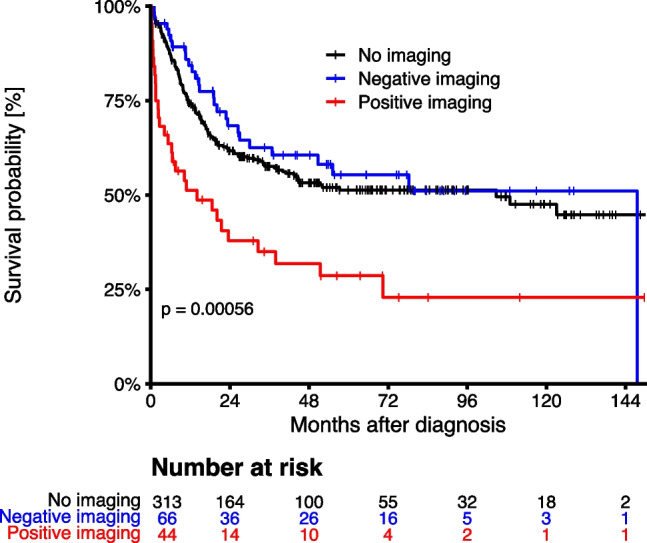
Overall survival. Overall survival of AML patients with acute new pathological findings (“positive imaging”) or without acute pathologies (“negative imaging”) on cranial imaging (CI) during intensive induction or consolidation chemotherapy. Also shown are AML patients that had not received any CI. P value by log–rank test, comparing overall survival between patients with positive and negative imaging

Overall survival (OS) in AML patients with a positive finding on CI (14 months) was significantly worse compared to patients with negative imaging (148 months) (Fig. 2). In patients with negative CI, overall survival was similar to the group of AML patients that had not received any CI (Fig. 2). A multivariate Cox regression analysis with age and ELN2010 risk group as additional covariates confirmed positive imaging as an independent risk factor for death in AML patients (Table 6).

**Table 6:** Multivariate analysis of risk factors for death

Risk factor	Comparator	HR	95% CI	*P* value
Positive imaging	Negative imaging	2.54	(1.41–4.55)	0.002
Age ≥ 65 years	Age < 65 years	2.68	(1.52–4.73)	< 0.001
ELN2010: 2	ELN2010: 1	1.25	(0.58–2.71)	0.56
ELN2010: 3	ELN2010: 1	1.16	(0.49–2.75)	0.74
ELN2010: 4	ELN2010: 1	2.66	(1.11–6.38)	0.03

Cox proportional hazard regression analysis

*ELN2010*, AML risk classification according to the European LeukemiaNet 2010 score; *HR*, hazard ratio; *CI*, confidence interval

## Discussion

Our single-center retrospective study systematically describes indications and associated clinical findings, diagnostic yield, and outcomes of CI in AML patients admitted for intensive induction or consolidation chemotherapy. In our study collective, 40% of all CI yielded an acute new, positive finding of which the majority was judged as a major (potentially severe) finding. Most CI used non-contrast-enhanced CCT. Positive imaging was associated with worse overall survival. Interestingly, AML patients with a negative first imaging exhibited an OS similar to AML patients without any imaging, indicating sufficient sensitivity of the chosen imaging modality to detect major pathologies.

We had previously investigated risk factors specifically for intracranial hemorrhage in AML patients receiving induction chemotherapy that were assessable at initial diagnosis [14]. Here, we comprehensively evaluated all patients undergoing induction or consolidation chemotherapy who had received CI. WHO classification and AML risk groups (ELN2010) were not associated with particular imaging findings. However, female gender and older age were found to be associated with acute pathological findings upon CI. In agreement with this, an association between female gender [14, 17] as well as increased age [14] and intracranial hemorrhage in AML patients had been previously found, although the underlying etiology remains unclear.

Of note, we failed to detect a statistically significant association between any particular indication for CI or laboratory values at time of imaging and a positive imaging result. This may be due to insufficient statistical power; however, it may also be a result of a low threshold for imaging combined with non-standardized clinical assessment. Due to the lack of clearly discernible associations, we have not performed further multivariate risk analyses. Every attempt was made to extract all available information describing patient presentation, preceding events and neurological symptoms at time of imaging from the records; nevertheless, not all information was available in every case. In obvious cases of newly onset focal neurology, the indication for imaging is clear; however, AML patients generally presented with variable history, complaints, and symptoms.

AML patients are at risk for intracranial hemorrhage due to thrombocytopenia and platelet dysfunction. In our cohort, intracranial hemorrhage (ICH) constituted the most frequent acute new finding in CI and this led to a change in therapy in all patients. The second most frequent new finding was acute sinusitis. However, this finding was of questionable clinical relevance due to the very low threshold for empirical anti-infective therapy in AML patients. Data regarding the impact of non-fungal sinusitis in this context is scarce [18]. No cases of acute invasive fungal sinusitis were diagnosed using CI, and we did not note any therapy changes due to an CI finding of acute uncomplicated sinusitis. The diagnosis of non-fungal, uncomplicated sinusitis by CI therefore seems to be a mostly benign finding in AML patients.

AML patients are clearly at a high risk of developing potentially severe intracranial acute pathologies, and the clinical variability is a challenge for the formulation of a tentative diagnosis and decision making for CI: although standardized criteria for CI in AML patients may be desirable, it appears unlikely that this can be achieved in a comprehensive manner based on the limited data published so far or presented here. A systematized approach may optimize pre-test probabilities. However, until more data is available, good clinical judgement, interdisciplinary clinical assessment, and a low threshold for CI in case of suspicion for cranial pathologies with potentially atypical presentation appear to be the optimal general strategy for timely diagnosis.

Our study has several important limitations, and we acknowledge the limited predictive value of a single-center retrospective explorative analysis. Our results require independent confirmation. The heterogeneity in clinical symptoms further limits the conclusions that can be drawn from data. A low threshold for CI (available inhouse 24/7) was maintained; however, there were no standardized criteria established to trigger imaging. Additionally, we do not have standardized longitudinal clinical assessments available for AML patients and examinations were in most cases not performed by trained neurologists. Further, the imaging reports had not been centrally validated by an independent reader. Therefore, it is not possible to evaluate the positive predictive value of inhospital falls or specific clinical findings and our results may not readily generalize to other hospital settings.

In summary, this study shows that CI in AML patients has a high diagnostic yield. Our data suggests to order CI at a low threshold of clinical suspicion because AML patients appear to have highly variable clinical presentation of intracranial pathologies. Incidental uncomplicated sinusitis diagnosed by CI is frequent, but adverse consequences were not noted.

## Data Availability

The data that support the findings of this study are not publicly available due to data protection restrictions but are available from the corresponding author on reasonable request.
